# Assessment of Hematological Parameters in Malaria, among Adult Patients Attending the Bamenda Regional Hospital

**DOI:** 10.1155/2020/3814513

**Published:** 2020-04-21

**Authors:** Nfor Omarine Nlinwe, Tang Bertilla Nange

**Affiliations:** The University of Bamenda, Faculty of Health Sciences, Department of Medical Laboratory Science, PO Box 39 Bambili, Bamenda, Cameroon

## Abstract

Malaria, which is responsible for a substantial amount of deaths in endemic countries, has been shown to have both direct and indirect effects on the hematological parameters. Notwithstanding, some hematological parameters among populations living in malaria endemic regions have not been described consistently, as a standard for measuring malaria burden. Based on the above fact, this study was designed to assess some hematological changes and their diagnostic values in malaria infected patients. A total of 160 malaria positive adult patients, together with 81 healthy control adults were recruited for the study. For the malaria positive group, the female to male ratio was 1.38 : 1. Specifically, 74.38%, 10.00%, and 15.62% of those in the malaria positive group had mild, moderate, and severe parasitaemia, respectively. Leukemia, anemia, and thrombocytopenia were found to be significantly associated with malaria and were all estimated to be specific for the diagnosis of malaria. Anemia was, however, estimated to be both sensitive and specific for malaria diagnosis. Therefore, anemia offers the most diagnostic value in the malaria infected patients of this study.

## 1. Introduction

Global malaria cases reduced from the year 2017 (estimated 219 million cases) compared to what was reported in 2010 (estimated 239 million cases) [1]. However, there was no significant progress in the reduction during 2015–2017 [1]. Consequently, malaria is still responsible for a substantial amount of deaths in endemic countries, especially in sub-Sahara Africa [2, 3]. In the WHO African Region, the dominant malaria parasite *Plasmodium falciparum* accounts for up to 99.7% of malaria cases in 2017 [1]. In these tropical regions, other common febrile illnesses show nonspecific overlapping signs and symptoms, causing a challenge to the clinical diagnosis of malaria [4]. For these reasons, the indiscriminate use of antimalaria is highly promoted [5, 6]. Moreover, the World Health Organization (WHO) recommends the use of antimalarial drugs based on a definitive demonstration of parasites in the peripheral blood film [7, 8]. Therefore, in cases of low malaria parasitaemia, certain automated hematological parameters could prompt peripheral blood smear examination for parasitic forms [5, 9–11]. Since malaria parasites are blood parasites, hematological changes are the most common complications encountered [12, 13]. They, therefore, account for some of the major role players in malaria pathology [14–17]. Despite its direct and indirect consequence on malaria, some hematological parameters have not been described consistently as a standard for measuring malaria burden [1]. In order to specifically diagnose acute febrile illnesses caused by malaria, a diagnostic clue using hematological parameters can be useful. Such routine laboratory findings include values of hemoglobin, leukocytes, platelet counts, and red cell distribution width [18]. Therefore this study was designed to improve the diagnostic specificity and the quality of care for patients with both malarial and nonmalarial fevers. Specifically, this study seeks to assess some hematological changes and their diagnostic values in malaria infected patients.

### 1.1. Background Literature

Anemia, as well as alterations in other hematological factors, is shown to be generally affected by malaria [19–21]. This is particularly dangerous because recurrent malaria incidents can cause life threatening anemia and metabolic acidosis, especially in children [22, 23]. Compared to children without malaria, children with malaria had significantly lower platelet counts [24]. The prevalence of anemia among malaria positive children was higher than those who were malaria negative [19]. In malaria infected children in western Kenya, platelets, lymphocytes, eosinophils, red blood cell count, and hemoglobin (Hb) were found to be significantly lower [15]. Meanwhile absolute monocyte and neutrophil counts and mean platelet volume (MPV) were higher in comparison to nonmalaria infected children [15]. Children with platelet counts of <150,000 *μ*L were 13.8 times (odds ratio) more likely to have malaria [15]. Similarly, malaria was reported as one of the causes of pancytopenia (i.e., hemoglobin < 10 g/dL, absolute neutrophil count < 1.5 × 10^9^/L and platelet count < 100 × 10^9^/L) in pediatric age groups [20]. A study carried out among children in North-Western Nigeria reveals a significantly higher occurrence of thrombocytopenia and anemia among subjects parasitized with *Plasmodium*, compared to nonparasitized controls [21].

Hematological factors are equally affected by malaria in adults as well as in pediatric groups. In a study where blood cells and platelets in *Plasmodium falciparum* malaria infection were evaluated, changes in white blood cells were found to be less severe than thrombocytopenia [25]. Thrombocytopenia typically disappeared with the treatment of the disease [25]. In another study, it was also observed among malaria patients that the frequency of alteration in hemoglobin and platelet counts was more prominent, compared to WBC counts [26]. Also, red blood cells (RBCs) count, hemoglobin (Hb), platelets count, white blood cells (WBCs) count, neutrophil, monocyte, lymphocyte, and eosinophil counts were found to be significantly lower in malaria infected patients [27].

In severe malaria, some of the hematological alterations differed with the developed complications [23]. Other findings show that, unlike platelet and white blood cell counts, hemoglobin concentration was found to be significantly different among the various complications of severe malaria [23]. However, in other studies, the following hematological abnormalities regularly accompanied infection with malaria: anemia, thrombocytopenia, splenomegaly, leucopenia, leukocytosis, mild-to-moderate atypical lymphocytosis, and rarely disseminated intravascular coagulation [18, 28]. Sen et al. reported a lesser degree of parasitaemia in those with chronic *Plasmodium falciparum* malaria [29]. Notwithstanding anemia, neutropenia, lymphocytosis, monocytosis, and thrombocytopenia were more severe in those patients with chronic *Plasmodium falciparum* malaria, as compared to those with acute *Plasmodium falciparum* malaria [29]. However, thrombocytopenia was reported to be an early sign of malaria infection, especially in *Plasmodium falciparum* malaria [30]. Among children, low hemoglobin concentration and platelet count were the most important forecasters of malaria infection [15]. There was a direct correlation between the ratio of monocytes to lymphocytes and the risk of clinical malaria among children with asymptomatic *Plasmodium falciparum* infection was higher [31]. As reported in another study, low lymphocyte counts, WBCs, and platelets were the most important predictors of malaria infection [27]. Many other studies also reported that thrombocytes, leucocytes, and RBCs are the major cell types known to be affected by malaria infection [15, 23, 31]. Consequently, hematological parameters could contribute to the determination of the malaria burden. This is especially useful, with the absence of a differential diagnostic test that could be used to differentiate between malaria sickness and parasitaemia with a concomitant fever of a different cause [32]. In endemic countries, there are complications with malaria diagnosis because individuals with partial immunity to malaria may be parasitized but not ill, or be ill from another disease [32]. For the patients who are parasitized and get sick of severe malaria, microscopy is the preferred method to monitor response to treatment because it can provide a quantitative evaluation of parasitaemia [33]. However, in resource-poor settings, there is high variability in the specificity and sensitivity of the microscopy method, posing a challenge in the provision of quality microscopy services [6]. The sensitivity of rapid diagnostic tests (RDTs) is equally greatly determined by factors such as the antigen, malaria species targeted, and the specific product used [34, 35].

## 2. Materials and Methods

### 2.1. Study Area and Population

This study was carried out in the Bamenda Regional Hospital (BRH), the chief government hospital in the North West Region of Cameroon. Known to be one of the ten regional headquarters in Cameroon, Bamenda is located along 10.15 longitude and 5.96 latitude. Bamenda is also situated at a height of 1258 meters above sea level. There are two seasons in Bamenda, the dry and the rainy seasons, with a balanced rainfall per year being 2064 mm (and 172 mm per month). The peak of dry season occurs in January. Meanwhile, the peak of the rainy season is in September. The BRH is part of the Bamenda Health District (BHD), which is made up of many public, private, and mission health facilities located within the 17 health areas in the BHD. The BRH, therefore, functions as the referral hospital in the region, with an estimated 337,036 inhabitants [36].

### 2.2. Study Participants/Study Period

This study was carried out within a period of three months, from February 2018 to April 2018. The inclusion criteria for the study were all adult (≥18 years) patients who tested malaria positive by the microscopic method. However, healthy malaria negative adults were included in the study as the control group. All those with an established diagnosis of systemic infections, typhoid fever, and meningitis were excluded from the study.

### 2.3. Ethical Consideration

The ethical clearance for this study was gotten from the Ethical Review Committee of the University of Bamenda. Signed informed consent was acquired from those who accepted to be enrolled in the study.

### 2.4. Sample Collection

Approximately 2-3 mL of venous blood samples were collected into EDTA anticoagulated test tubes, from patients who were sent to the BRH laboratory for a malaria test. Blood films (thick and thin) were prepared within a period of 30 minutes, following the techniques recommended by Cheesbrough [37].

### 2.5. Microscopy Test Method

The prepared blood films were processed and stained with 3% Giemsa staining technique [37]. Two experienced microscopists independently examined duplicate slides. A third experienced microscopist confirmed results with discrepancies. Parasite density per microlitre of blood was estimated following the methods in a previous study [38]. The cut-off level for mild parasitaemia was <1,000 parasites/*μ*L of blood, moderate parasitaemia was 1,000 to 9,999 parasites/*μ*L of blood, and severe parasitaemia ≥10,000 parasites/*μ*L of blood. This was based on a previous study by Sumbele et al. [39].

### 2.6. Blood Cells Count Analysis

All malaria positive blood samples and those of the control group were analyzed for blood cell counts. Blood counts were performed using an automated hematology analyzer, the Beckman Coulter counter (URIT-3300), following the manufacturer's instructions. The following hematological parameters were considered for this study: white blood cell counts (WBC), percentage of lymphocytes (LYM%), percentage of monocytes (MON%), percentage of neutrophils (NEUT%), hemoglobin measurements (Hb), red blood cell distribution width (RDW-CV), and platelet counts (PLT).

### 2.7. Data Analysis

Baseline characteristics of hematological parameters in patients with and without malaria were determined using the statistical package Stata Software version 12.14. These baseline characteristics include the range, mean, and standard deviation, all at 95% confidence interval. A fourfold (2 × 2) contingency table displaying the frequency distribution for each hematological parameter was entered into GraphPad Prism version 8.2.1. In each of the four cells, the contingency table had frequencies for normal and abnormal hematological values of both the positive and negative malaria cases. Chi-square (and Fisher's exact) test was used to calculate sensitivity (%), specificity (%), predictive values (%), likelihood ratios, odds ratios, relative risk, and attributable risk. The level of reliability of the results was determined by the confidence interval, which in this study was at 95%. Frequencies of hematological parameters with statistical significance were further combined and analyzed in contingency tables as described above. This was done to assess the association between combined hematological variables and malaria status.

## 3. Results

All the malaria positive cases were *Plasmodium falciparum* species.  Table 1 shows the distribution of the degree of malaria parasitaemia according to sex. A greater percentage (74.38%) of those infected suffered from mild parasitaemia; meanwhile 10% (16/160) and 15.62% (25/160) suffered from moderate and severe parasitaemia, respectively. The positive malaria group was composed of 93 females and 67 males, with a female to male ratio being 1.38 : 1. The majority of those with mild (63.87%) and moderate (68.75%) parasitaemia were females, whereas the majority of those with severe parasitaemia (76%) were males.

The mean hematological values, confidence intervals, and *P* values of the hematological parameters in those with and without malaria are shown in Table 2 below. There was a statistically significant reduction in white blood cell counts (*P* < 0.0001), HGB levels (*P*=0.0006), and platelet counts (*P*=0.0164) in those with malaria as compared to those without malaria. There were no significant differences between the mean values of LYM%, MON%, NEUT%, and RDW_CV, in patients with malaria, compared to those without malaria.

With 95% confidence interval, the sensitivity, specificity, likelihood ratio, and positive and negative predictive values were estimated for the diagnosis of malaria using the hematological parameters. Statistically, the sensitivity of leucopenia (WBC < 4 × 10^3^/*μ*L)-26.67%, monocytopenia (MON% < 1%)-0% and thrombocytopenia (<100 × 10^3^/*μ*L)-23.48% were all significantly low. But anemia (Hb < 12 g/dL) had a statistically significant sensitivity of 68.12%. However, leucopenia (WBC < 4 × 10^3^/*μ*L), monocytopenia (Mon% < 1%), and thrombocytopenia (PLT < 100 × 10^3^/*μ*L) had significantly high specificity of 94.92%, 95.06%, and 90.63%, respectively. Anemia (Hb < 12 g/dL) had a significant specificity (63.64%). The sensitivity and specificity for lymphopenia (lym% < 20%), neutrophilia (Neut% ˃ 70%), and low RDW_CV (RDW_CV < 11.5%) were not statistically significant. The likelihood ratio for the diagnosis of malaria using leukopenia, anemia, and thrombocytopenia was 5.244, 1.873, and 2.505, respectively.

A combination of positive tests for the following hematological parameters was carried out: WBC < 4 × 10^3^/*μ*L, Mon% < 1%, PLT < 100 × 10^3^/*μ*L, and Hb < 12 g/dL (Table 3). This combination was thought necessary since all tests are done simultaneously, using the hematological analyzer. The sensitivity, specificity, predictive values, and likelihood ratio were obtained with the combined hematological parameters along with 95% confidence intervals for the diagnosis of malaria. The combined likelihood ratio (3.429) for the two variables, WBC < 4 × 10^3^/*μ*L and PLT < 100 × 10^3^/*μ*L, was the highest, followed by the combined likelihood ratio (2.149) for the three variables: WBC < 4 × 10^3^/*μ*L, Hb < 12 g/dL, and PLT < 100 × 10^3^/*μ*L.

The odds ratio, relative risk, and attributable risk of the hematological parameters for the diagnosis of malaria, at 95% confidence interval, are shown in Table 4. WBC < 4 × 10^3^/*μ*L, Hb < 12 g/dL, and PLT < 100 × 10^3^/*μ*L, are the hematological parameters with the highest odds ratios of 6.788, 3.739, and 2.967, respectively. These parameters also had the highest relative risk values of 1.445 (WBC < 4 × 10^3^/*μ*L), 1.629 (HGB < 12 g/dL), and 1.319 (PLT < 100 × 10^3^/*μ*L). Their values for the attributable risk were equally among the highest: 0.2844 (WBC < 4 × 10^3^/*μ*L), 0.2974 (HGB < 12 g/dL), and 0.2026 (PLT < 100 × 10^3^/*μ*L).

### 3.1. Discussion of Results

Among the malaria positive cases, up to 74.38% (119/160) had mild parasitaemia; meanwhile, only 10% (16/160) had moderate parasitaemia, and 15.62% (25/160) had severe parasitaemia. In line with expectation, mild parasitaemia was most prevalent among the study subjects who were all adults. Studies have observed that in malaria endemic areas, immunity against malaria develops with time [40]. Therefore at adulthood, the majority of malaria infections may probably be mild and asymptomatic [40]. Hence, individuals who appear healthy may carry the parasite in their blood. In fact, it was reported that malaria complications are most prevalent among individuals who are parasitized without illness [32]. Nonetheless, this was not the case with this study. In the current study, all malaria positive cases were symptomatic patients attending the Bamenda Regional Hospital. Although parasitaemia was mostly mild, the patients were, however, symptomatic. This agrees with the report by WHO, 2000, on “Severe falciparum malaria.” It was reported that persons with mild malaria normally present clinically with fever [41]. Since cases with low parasitaemia could be missed during peripheral blood smear examination, changes in certain hematological parameters can prompt a more careful microscopic examination for malaria diagnosis.

In this study, up to 63.87% (76/119) of those with mild and 68.75% (11/16) of those with moderate parasitaemia were females. But 76.00% (19/25) of those with severe parasitaemia were males. There was a higher parasite density in males. Since the economy of the study area has its man power highly concentrated in the primary sector, the men mostly work out doors throughout the day. But the women rather rush home to focus on house chores during the dusk period of the day. Therefore, the men become more exposed to mosquito bites, increasing their chances of being repeatedly infected with malaria. Malaria infections were also reported to be more severe in males compared to females, by other authors [42, 43]. According to these authors, gender differences in exposure patterns also accounted for the increased levels of parasite density in males as compared to females. Similar findings were reported in mice. A study suggested that estrogen may influence differential transcription and translation of IFN-*γ*, causing gender differences in response to malaria [44]. In that study, there was reduced mortality and a faster rate of recovery among female mice as compared to males [44]. Likewise, testosterone was found to mediate the suppression of resistance against blood-stage malaria in mice [45]. However, hormonal influence on malaria severity is yet to be confirmed in humans [46]. In contrast with findings from the current study, a study that sampled 50 malaria positive cases reported a higher prevalence in females (56%) as compared to males (44%) [47]. The difference was not, however, classified according to the levels of parasitaemia and moreover, the smaller sample size in this study may have accounted for this discrepancy. Notwithstanding, men and women may have equal risks of infection depending on their activities in the peak biting periods. In the current study, the clear cut differences in parasite density between male and female are possibly mediated by differences in exposure patterns.

As shown in Table 5, there was a statistically significant (*P*=0.0004) reduction in WBC count in those with malaria, as compared to those without malaria. This finding agrees with other findings where WBC counts, among other hematological parameters, were found to be significantly lower in malaria infected patients [15, 16, 31]. However, findings from other sources reveal that compared to alterations in other hematological parameters like platelet counts and hemoglobin levels, changes in WBC counts were less severe [25, 26]. In any case, WBC counts in the present study have been shown to assist in the determination of malaria burden. Although the sensitivity (26.67%) and NPV (36.13%) of leucopenia (WBC < 4 × 10^3^/*μ*L) were significantly low, it had a high specificity (94.92%) and PPV (92.31%) for the diagnosis of malaria. Consequently, findings from this study suggest that although leucopenia do not necessarily signify a positive malaria test (because of low sensitivity), there is a higher probability that those without leukopenia will test negative for malaria. Relative to the other hematological parameters, leukopenia also had the highest likelihood ratio (5.24) for malaria diagnosis. This implies that those with leukopenia may have a higher chance of testing malaria positive. The odds ratio (6.788) for leukopenia is very high, also indicating that those with leukopenia have a higher chance of testing positive for malaria. Additionally, the relative risk of 1.445 indicates that the study participants with leukopenia have a 44.5% higher risk of testing positive for malaria than those without leukopenia.

There was equally a statistically significant reduction in hemoglobin levels (HGB < 12 g/dL) in those with malaria, in comparison to those without malaria. Anemia in malaria infected children and adults have been frequently observed over the years [15, 19–21, 26, 27]. Hemoglobin concentrations were even found to be significantly different among various complications of severe malaria among adults in Aden [23]. This is not strange as rapid recovery from malaria anemia is compromised by malaria induced hemolysis of both the infected and noninfected red blood cells, in addition to bone marrow dyserythropoiesis. In fact, anemia and low platelet counts were projected as the most important forecasters of malaria infection [15]. In line with findings from this study, the sensitivity (68.12%), specificity (63.64%), PPV (77.05%), and NPV (52.69%) of anemia, for the diagnosis of malaria, could be considered high for most cases. Unlike the case of leukopenia which had low sensitivity for malaria diagnosis, there is a higher probability that anemic individuals will be diagnosed positive with a malaria test. Moreover, the high specificity indicates that the absence of anemia will likely indicate a malaria negative test. Likewise, the likelihood ratio of 1.873 confirms the fact that anemia in malaria endemic area could feasibly be used as a diagnostic tool for malaria diagnosis. Additionally, the odds ratio for anemia being used as an indicator for the presence of malaria is 3.739. This greatly deviates from the average of 1, signifying that among those with anemia, the outcome of being malaria positive is more than 100% likely. Moreover, the relative risk of 1.629 shows that those with anemia had a 62.9% higher risk of testing malaria positive. However, in other findings among children (<5 years) who were exposed to between 1 and 2 years of malaria control, mean relative risk for Hb < 11 g/dL was 0.73 (95% CI 0.64–0.81) and 0.40 (95% CI 0.25–0.55) for hemoglobin < 8 g/dL [48–50]. These are comparatively better than what was gotten in the current studies, probably because the study participants in this study were exposed to some years of malaria control.

As shown in Table 2, platelet counts were significantly (*P*=0.0164) reduced in malaria positive cases, in comparison to those without malaria. In line with studies among malaria infected children in western Kenya, platelets, alongside other hematological parameters, were found to be significantly lower [15]. The significant occurrence of thrombocytopenia in malaria positive cases has also been reported in several other studies [18, 20, 21, 25, 27, 28]. This may likely be caused by the destruction of platelet by macrophages, bone marrow alterations, antibody-mediated platelet destruction, oxidative stress, and platelet aggregation, coagulation disturbances and splenomegaly, which are all related to the pathogenesis of malaria thrombocytopenia [51, 52]. Thrombocytopenia was observed to be more frequent, relative to leucopenia and anemia [26], and has also been reported as an early sign of malaria infection [30]. Similar findings were recorded from a recent study, including 200 adult malaria patients [22]. Findings from this study suggest thrombocytopenia as a possible risk factor for severe malaria. The study concluded that thrombocytopenia and anemia can be used as diagnostic standards in predicting severe *Plasmodium falciparum* malaria in adults [22].

As shown in Table 5 above, although the sensitivity of thrombocytopenia (23.48%) was significantly low, its specificity (90.63%) for malaria diagnosis was significantly high. As a result, even though thrombocytopenia may not reliably indicate malaria positivity, individuals with normal platelet counts will most likely have a malaria negative test results. Moreover, the likelihood ratio of 2.505 further indicates that those with thrombocytopenia will most likely be malaria positive, as compared to those without malaria. The odds ratio of thrombocytopenia being 2.967 equally indicates that study participants with low platelet counts are more than 100% likely to test positive for malaria, as compared to those without. The relative risk of 1.319 further indicates that those with thrombocytopenia are at a 31.9% higher risk of testing malaria positive.

The probability of having a positive malaria test was increased when the hematological variables with significant change (Table 3) were combined. The LR for all four, three, and two variables combined were all greater than 1, indicating a higher probability of their association with a malaria positive test. However, the LR of leukopenia alone (5.244) was higher than that of anemia and thrombocytopenia combined (3.429). But Dhangadamajhi et al. found that anemia and thrombocytopenia can be used in combination to predict malaria infection in adults [22].

The absence of significant differences between the mean values of LYM% in patients with malaria compared to those without malaria contradicts other findings where mild atypical lymphocytosis was found to accompany malaria [18, 28]. However, these discrepancies may be insignificant since lymphocytosis was just mild. However, in another study, low lymphocyte counts were among the most important prediction of malaria [27]. The absence of significant differences between the mean values of LYM%, MON%, NEUT%, and RDW-CV in this study could be generally explained by the fact that parasitaemia was mostly mild.

## 4. Conclusion

This study which sought to assess some hematological changes and their diagnostic values in malaria infected patients found out that hemoglobin levels, white blood cell counts, and platelet counts were significantly reduced among the malaria positive individuals. Based on this outcome, it is recommended that in malaria endemic areas predominated by mild parasitaemia, leukemia, thrombocytopenia, and anemia could be used as prognosticators for infections with malaria. This recommendation can be more appropriate for the adult group.

## Figures and Tables

**Table 1:** Distribution of malaria parasitaemia according to sex.

Distribution	Mild parasitaemia (%)	Moderate parasitaemia (%)	Severe parasitaemia (%)	Total cases (%)
Total cases (%)	119 (74.38)	16 (10)	25 (15.62)	160
Male	43 (36.13)	5 (31.25)	19 (76.00)	67 (41.88)
Female	76 (63.87)	11 (68.75)	6 (24.00)	93 (58.12)

**Table 2:** Baseline characteristics of hematological parameters in patients with and without malaria.

Parameters	Expected range	With malaria (*N* = 160)		Without malaria (*N* = 81)		*P* value
		Mean (SD)	95% CI	Mean (SD)	95% CI	
WBC	4.0–10.0	7.391 (5.24)	6.573–8.208	9.515 (10.96)	7.091–11.94	<0.0001^*∗*^
LYM%	20.0–40.0	29.6 (22.59)	26.08–33.13	30.31 (15.07)	26.98–33.64	0.8901
MON%	1.0–15.0	6.42 (2.30)	6.06–6.78	7.00 (2.98)	6.34–7.66	0.1216
NEUT%	50.0–70.0	64.4 (16.66)	61.8–67	62.73 (17.07)	58.95–66.5	0.4165
HGB	12.0–16.0	11.29 (2.50)	10.89–11.68	12.09 (3.11)	11.4–12.77	0.0006^*∗*^
RDW_CV	11.5–14.5	12.51 (1.70)	12.25–12.78	13.5 (2.41)	12.96–14.03	0.0781
PLT	100–300	207.4 (127.6)	187.5–227.4	238.5 (113)	213.5–263.4	0.0164^*∗*^

^*∗*^Significant *P* value.

**Table 3:** Combination of hematological parameters (WBC, Mon%, HGB, Platelets) for malaria diagnosis.

Combined variables	Sensitivity	Specificity	PPV	NPV	LR	*P* value
Wbc < 4, Mon% < 1 and/or ˃ 15, HGB < 12, platelet < 100	(95% CI)	(95% CI)	(95% CI)	(95% CI)	(95% CI)	
All four	28.5	85.41	79.7	37.27	1.953	<0.0001
	24.93 to 32.35	80.80 to 89.06	73.63 to 84.67	33.62 to 41.07		
Any three positive	39.75	81.5	81.31	40.05	2.149	<0.0001
Wbc < 4, HGB < 12, platelet < 100	35.10 to 44.59	75.54 to 86.27	75.31 to 86.13	35.40 to 44.88		
Any two	47.62	77.21	80.75	42.34	2.089	<0.0001
Wbc < 4, HGB < 12	41.77 to 53.54	69.47 to 83.45	73.97 to 86.09	36.35 to 48.56		
Any two	25.09	92.68	88.16	36.31	3.429	<0.0001
Wbc < 4, platelet < 100	20.27 to 30.62	86.68 to 96.10	79.00 to 93.64	31.18 to 41.76		
Any two	46.3	75.89	78.62	42.46	1.92	<0.0001
HGB < 12, platelet < 100	40.44 to 52.25	68.20 to 82.20	71.61 to 84.27	36.52 to 48.63		

**Table 4:** The odds ratio, relative risk, and attributable risk of the hematological parameters for the diagnosis of malaria.

Variables	Odds ratio	Relative risk	Attributable risk
	(95% CI)	(95% CI)	(95% CI)
Wbc < 4	6.788	1.445	0.2844
	2.110 to 21.68	1.214 to 1.674	0.1234 to 0.3837

Lym% < 20	0.9747	0.9924	0.005357
	0.5076 to 1.841	0.8054 to 1.199	−0.1415 to 0.1437

Mon% < 1 and/or ˃ 15	0	0	0.6751
	0.000 to 0.5015	0.000 to 0.7285	0.6121 to 1.283

Neut% ˃ 70	1.219	1.062	0.04191
	0.6628 to 2.245	0.8851 to 1.285	−0.08989 to 0.1740

HGB < 12	3.739	1.629	0.2974
	2.042 to 6.527	1.304 to 2.090	0.1607 to 0.4219

RDW_CV < 11.5	0.7247	0.8715	0.07861
	0.3725 to 1.432	0.6235 to 1.149	−0.09484 to 0.2504

Platelet < 100	2.967	1.319	0.2026
	1.188 to 7.185	1.058 to 1.562	0.02221 to 0.3267

**Table 5:** Sensitivity, Specificity, Predictive values, and Likelihood ratio (LR) for the diagnosis of malaria with the hematological parameters.

Variables	Sensitivity (%)	Specificity (%)	PPV (%)	NPV (%)	LR	*P* value
	(95% CI)	(95% CI)	(95% CI)	(95% CI)	(95% CI)	
Wbc < 4 × 10^3^/*μ*L	26.67	94.92	92.31	36.13	5.244	0.0004^*∗*^
	19.93 to 34.70	86.08 to 98.61	79.68 to 97.35	0.2899 to 0.4394		

Lym% < 20%	38.28	61.11	70	29.46	0.9844	>0.9999
	30.32 to 46.93	47.79 to 72.96	58.46 to 79.46	21.82 to 38.47		

Mon% < 1% ˃ 15%	0	95.06	0	32.49	0	0.0121^*∗*^
	000 to 2.345	87.98 to 98.06	0 to 48.99	26.85 to 38.69		

Neut% ˃ 70%	54.93	50	71.56	32.63	1.099	0.5443
	46.72 to 62.88	37.92 to 62.08	62.47 to 79.18	24.04 to 42.57		

Hb < 12 g/dL	68.12	63.64	77.05	52.69	1.873	<0.0001^*∗*^
	59.94 to 75.31	52.48 to 73.49	68.83 to 83.62	42.64 to 62.53		

RDW-CV < 11.5%	22.64	71.23	53.33	38.81	0.7871	0.3838
	15.71 to 31.48	59.99 to 80.35	39.08 to 67.06	30.98 to 47.26		

Platelet < 100 × 10^3^/*μ*L	23.48	90.63	83.78	36.48	2.505	0.0194^*∗*^
	17.07 to 31.40	81.02 to 95.63	68.86 to 92.35	29.40 to 44.20		

^*∗*^Significant *P* value.

## Data Availability

All the data for this study can be acquired from the authors upon request.
